# Evaluation of left ventricular systolic function in patients with different types of ischemic heart disease by two-dimensional speckle tracking imaging

**DOI:** 10.1186/s13019-020-01345-2

**Published:** 2020-11-04

**Authors:** Xing Xing, Dan Li, Shaomin Chen, Lingli Wang, Zhaoping Li, Liyun He

**Affiliations:** Department of Cardiology and Institute of Vascular Medicine, Peking University Third Hospital, NHC Key Laboratory of Cardiovascular Molecular Biology and Regulatory Peptides, Key Laboratory of Molecular Cardiovascular Science, Ministry of Education, Beijing Key Laboratory of Cardiovascular Receptors Research, 49 North Garden Rd, Haidian District, Beijing, 100191 China

**Keywords:** 2D-STI, Left ventricular systolic function, Ischemic heart disease

## Abstract

**Background:**

The purpose of this study was to evaluate left ventricular systolic function in patients with different types of ischemic heart disease using two-dimensional speckle tracking imaging (2D-STI).

**Methods:**

We retrospectively studied patients who were admitted to Peking University Third Hospital from January 2011 to December 2017 due to chest tightness and chest pain. Two hundred forty-two patients were divided into control group, CMD group and obstructive CAD group. The main coronary artery stenosis was confirmed by coronary angiography or coronary computed tomography and coronary flow reserve (CFR) in patients was measured by transthoracic Doppler echocardiography. Left ventricular strain and strain rate (SR) measured by 2D-STI. Cardiac structure and function were measured by conventional echocardiography.

**Results:**

Conventional echocardiography showed that there was no significant difference in cardiac structure and function among the three groups (*P* > 0.05). Moreover, the longitudinal strain (LS) of each ventricular wall in CMD group was notably lower than that in control group (*P* < 0.01). In addition, global longitudinal SR and longitudinal SR in CMD group and obstructive CAD group were obviously lower than those in control group (*P* < 0.01). GLS, endocardial LS and epicardial LS were negatively correlated with CFR (*P* < 0.01).

**Conclusions:**

Early left ventricular systolic dysfunction was found in patients with CMD and patients with obstructive CAD, with similar degree. CFR is an independent influencing factor of GLS. GLS and stratified LS have certain diagnostic value for CMD.

## Introduction

Ischemic heart disease is the leading cause of death in patients with cardiovascular disease [1]. Coronary obstructive atheromatous plaques have long been considered to be the main cause of myocardial ischemia [2]. However, in recent years, studies have found that patients with non-obstructive coronary heart disease (CAD) also have myocardial ischemia, and some patients with obstructive CAD still have myocardial ischemia after relieving coronary artery stenosis [3]. These evidences have led to a re-examination of the mechanism of IHD, which has gradually evolved from a pathological model centered on epicardial coronary atherosclerotic obstruction to ischemia at the microcirculation and cardiomyocyte levels.

Coronary microvascular dysfunction (CMD) and obstructive CAD can cause myocardial ischemia at the levels of myocardial microcirculation and epicardial coronary artery respectively, which can lead to abnormal cardiac structure and function and participate in the occurrence and development of heart failure [4, 5]. Obstructive CAD is one of the most common causes of heart failure [4]. It causes rational remodeling of heart disease through pathological mechanisms, such as myocardial ischemia, reperfusion injury and excessive activation of neurohormones, and impairs cardiac systolic and diastolic functions [6]. CMD is caused by many factors, such as microvascular remodeling, sparse vascular distribution, perivascular fibrosis, dysfunction of endothelial cells, dysfunction of smooth muscle cells, microvascular embolism, dysfunction of autonomic nervous system and extraluminal compression, which can result in coronary microvascular structure or dysfunction and insufficient effective myocardial perfusion [7]. Coronary flow reserve (CFR), the ratio of coronary flow to basal flow at maximum congestion, is often used to evaluate coronary microvascular function. CMD is an important pathological mechanism of heart failure with preserved ejection fraction (HFpEF), which can cause impaired diastolic function [8–10]. In theory, CMD can also cause the decline of cardiac systolic function through pathological mechanisms such as myocardial ischemia. However, there are limited studies on the effect of CMD on cardiac systolic function, and the conclusions are different [11, 12].

Two-dimensional speckle tracking imaging (2D-STI) calculates the motion ability of myocardial tissue by tracking the movement track of myocardial tissue speckles, so as to evaluate the systolic and diastolic function of heart. Compared with conventional echocardiography, STI can quantitatively evaluate cardiac motion and detect abnormal systolic function more sensitively. It is especially suitable for evaluating early damage of cardiac systolic function in patients whose left ventricular ejection fraction (LVEF) is still in the normal stage [13]. Strain and strain rate (SR) are the main indicators of myocardial contractility in STI measurement parameters. Strain is the percentage of changes in the length of myocardial fibers and SR is the change of strain per unit time. Longitudinal strain (LS) is often the earliest to be impaired in all directions of strain and is therefore more sensitive for evaluation of left ventricular systolic dysfunction [14, 15].

In this study, the strain and SR of Left ventricle in patients with CMD, patients with obstructive CAD and normal people were analyzed by 2D-STI, and the effects of myocardial microcirculation and epicardial coronary artery ischemia on cardiac systolic function were investigated.

## Subjects and methods

### Subjects

This retrospective single-centre study was based on the programme approved by the Institutional Review Committee of Peking University Third Hospital and was in accordance with the Helsinki Declaration. This study obtained approval from the Ethics Committee of our hospital and the informed consent rights from patients and their families. We retrospectively studied patients who were admitted to Peking University Third Hospital from January 2011 to December 2017 due to chest tightness and chest pain. Subsequently, the main coronary artery stenosis was confirmed by coronary angiography or coronary computed tomography and patients’ CFR was measured by transthoracic Doppler echocardiography. Exclusion criteria: (1) patients with history of myocardial infarction or abnormal wall motion suggested by conventional echocardiography; (2) patients with acute heart failure, chronic heart failure or LVEF values less than 50%; (3) patients with congenital heart disease, severe valvular disease, severe bradycardia, high atrioventricular block and atrial fibrillation; (4) patients who did not store ultrasound images or whose ultrasound images were unclear; (5) patients who had incomplete clinical data. After screening, there were 108 cases of patients with CFR ≥ 2.5 (control group), 67 cases of patients with CFR < 2.5 (CMD group) [16], and 67 cases of patients with major coronary artery stenosis > 50% and age-matched patients in CMD group (obstructive CAD group). All patients underwent routine echocardiographic examination within 1 week before coronary angiography, and echocardiographic images were preserved. The blood biochemical indexes in patients were measured by using BECKMAN AU5800 automatic biochemical analyzer and commercially available diagnostic kits within 1 week before coronary angiography.

### Measurement of left centricular strain and SR by 2D-STI

The patient was placed in the left decubitus position and connected to the electrocardiogram. A two-dimensional dynamic image of the apical two-chamber, three-chamber, and four-chamber long-axis sections was collected, and dynamic images of three consecutive cardiac cycles were stored. The recorded images were imported onto an EchoPAC ultrasound workstation for off-line analysis to determine left ventricular strain and SR. Under the condition of two-dimensional strain, the left ventricular endocardial boundary was manually delineated for each dynamic image at the end of systole. The software automatically generated the region of interest and adjusts its width to include the whole myocardium. After running the program, the software automatically tracked the myocardial motion in the region of interest from frame to frame and generated a “bull eye diagram”. Besides, the “Buffalo Eye Diagram” divided the left ventricular wall into 17 segments (3 for the anterior wall, lateral wall, posterior wall and posterior septum; 2 for the anterior septum and inferior wall, 1 for the apical cap), and showed LS value of each segment. Furthermore, the LS of apical two-chamber, three-chamber and four-chamber cardiac sections were measured. The mean value of LS of each section was left ventricular GLS. After selecting layered information, the system automatically divided GLS into subendocardial LS and subepicardial LS. After choosing the left ventricular SR information, the images showed 17 segments of longitudinal SR, and the mean value was left ventricular longitudinal SR. All measurements were averaged for three consecutive cardiac cycles.

### Routine echocardiography

Routine echocardiographic examinations of patients were performed by veteran echocardiographic ventricular physicians with Vivid E9 color Doppler echocardiography (GE company, USA). The parasternal left ventricular long axis section, aortic short axis section, left ventricular short axis section, apical four-chamber section and apical two-chamber section were taken to determine cardiac structure and function. We continuously record dynamic images of at least three cardiac cycles. Cardiac structural indicators included systolic left atrial anteroposterior diameter (LAD), interventricular septal thickness (IVST), left ventricular posterior wall thickness (LVPWT) and end-diastolic left ventricular diameter (LVEDD). Cardiac systolic function indicators included LVEF and systolic velocity of mitral annulus in left ventricular lateral wall (Sm). Cardiac diastolic function indexes included early peak mitral flow velocity (E), late peak mitral flow velocity (A), and early mitral annular motion velocity (E’) of left ventricular lateral wall diastolic. E/A and E/E’ were calculated respectively. Left ventricular mass was calculated according to Devereux formula.

### Measurement of CFR

The CFR of left anterior descending coronary artery was detected by Vivid E9 color Doppler echocardiography (GE company, USA) with probe frequency of 1.7/3.3 MHz. The drug that used to induce the maximum coronary hyperemia was (adenosine-triphosphate) ATP injection (Shanghai Harvest Pharmaceuticals Co., Ltd., Shanghai, China). Blood pressure (systolic blood pressure: SBP, diastolic blood pressure: DBP) and heart rate (HR) in patients were recorded at rest. Then, the patient took the left lateral position and the left ventricular short axis section near the apex of the heart between the fourth and fifth intercostals of the left sternum was displayed. The anterior interventricular sulcus between the left ventricle and the right ventricle was fully displayed by slightly counterclockwise rotation of the probe. After local enlargement, the distal blood flow of left anterior descending branch of coronary artery was detected by color Doppler echocardiography. After displaying the long axis of the vessel, the blood flow spectrum of the left anterior descending branch was measured by pulsed Doppler echocardiography and was recorded at rest. After fixing the probe, ATP (0.14 mg/kg·min) was continuously injected intravenously for 2 min and the blood flow spectrum of left anterior descending branch was continuously recorded. The peak diastolic flow velocity (PDV1) of left anterior descending artery in resting state and maximum peak diastolic flow velocity (PDV2) of left anterior descending artery after ATP injection were measured to calculate CFR. The calculation formula of CFR was as follows: CFR = PDV2 / PDV1.

### Statistical analysis

Software SPSS17.0 (International Business Machines, corp., Armonk, NY, USA) was used for statistical analysis. Counting data were expressed as frequency or percentage (n %). Chi-square test was used for comparison between groups. The measurement data were analyzed by normal test. The data with normal distribution were expressed as mean ± SD and the data with non-normal distribution were expressed as median (P25, P75). Mean comparison of continuous variables of normal distribution in three groups was used variance analysis and two-two comparison was used the LSD (L) method. Mean comparison of continuous variables of non-normal distribution in three groups was used Kruskal Wallis test and two-two comparison was used Mann-Whitney U test. Mean comparison of continuous variables of normal distribution between the two groups was used T-test. Correlation analysis of counting data with non-normal distribution was used Spearman correlation analysis. All tests were statistically significant with a bilateral *P* < 0.05.

## Results

### Basic clinical data of patients

The basic clinical data of the patients were shown in Table 1. A total of 242 patients were enrolled, including 102 males (42.1%) and 140 females (57.9%). Their ages ranged from 44 to 81 years, with an average age of (62.6 + 8.3) years. Among all the patients, 151 (62.4%) had hypertension, 56 (23.1%) had diabetes mellitus, 137 (56.6%) had dyslipidemia, and 60 (24.8%) had smoking. In addition, 31 patients had severe coronary artery stenosis (> 70%) and 36 patients had moderate coronary artery stenosis (50–70%). Furthermore, it showed that there were significant differences in age, history of diabetes mellitus, smoking history and taking antiplatelet drugs and statins among the three groups (*P* < 0.05 or *P* < 0.01). However, there were no significant differences in heart rate, SBP, DBP, BMI and taking other drugs (ACEI/ARB, CCB, β - blockers) among the three groups (*P* > 0.05).

**Table 1:** Comparison of basic clinical data of patients in obstructive CAD group, CMD group and control group

Parameters	obstructive CAD group (*n* = 67)	CMD group (*n* = 67)	control group (*n* = 108)	*P* value
Age (years old)	62.8 ± 9.0	64.9 ± 8.4	60.9 ± 7.4	0.007**
Male, n (%)	46 (68.7)	16 (23.9)	40 (37.0)	0.000**
BMI, kg/m^2^	25.3 ± 2.9	24.5 ± 3.2	25.1 ± 3.2	0.301
Hypertension, n (%)	43 (64.2)	48 (71.6)	60 (55.6)	0.096
diabetes mellitus, n (%)	25 (37.3)	12 (17.9)	19 (17.6)	0.005**
Dyslipidemia, n (%)	39 (58.2)	45 (67.2)	53 (49.1)	0.061
Smoke, n (%)	26 (41.9)	11 (19.0)	23 (20.9)	0.006**
HR, times/min	65.0 ± 8.5	65.2 ± 10.0	64.7 ± 7.7	0.917
SBP, mmHg	132.8 ± 14.4	132.4 ± 13.5	130.5 ± 13.0	0.473
DBP, mmHg	75.4 ± 10.7	73.4 ± 9.5	75.6 ± 9.1	0.311
antiplatelet drugs, n (%)	65 (97.0)	56 (83.6)	76 (70.4)	0.000**
Statin drugs, n (%)	60 (90.0)	55 (82.1)	80 (74.1)	0.030*
ACEI/ARB, n (%)	20 (30.0)	27 (40.3)	34 (31.5)	0.311
β-blockers, n (%)	33 (49.3)	24 (35.8)	40 (37.0)	0.247
CCB, n(%)	23 (34.3)	27 (40.3)	30 (27.8)	0.201

*BMI* body mass index, *SBP* systolic blood pressure, *DBP* diastolic blood pressure, *ACEI* angiotensin converting enzyme inhibitor, *ARB* angiotensin II receptor blockers, *CCB* calcium channel blocker. *: *P* < 0.05; **: *P* < 0.01

### Comparison of blood biochemical indexes in the three groups

The blood biochemical indexes of the patients were shown in Table 2. The levels of TC and HDL-C in obstructive CAD group were significantly lower than those in control group and CMD group (*P* < 0.05 or *P* < 0.01). In addition, the levels of LDL-C in obstructive CAD group were notably lower than those in normal group (*P* < 0.01), but there was no significant difference in obstructive CAD group and CMD group. However, the levels of TG in obstructive CAD group were obviously higher than those in CMD group (*P* < 0.01), but there was no significant difference in obstructive CAD group and control group. In addition, the levels of FBG and HbA1c in obstructive CAD group were significantly higher than those in control group and CMD group (*P* < 0.01). Moreover, the levels of UA and Cr in obstructive CAD group were obviously higher than those in control group and CMD group (*P* < 0.01). However, the levels of NTpro-BNP in CMD group were obviously higher than those in control group (*P* < 0.05), but there was no significant difference in obstructive CAD group and CMD group.

**Table 2:** Comparison of blood biochemical indexes in obstructive CAD group, CMD group and control group

Parameters	obstructive CAD group (*n* = 67)	CMD group (*n* = 67)	control group (*n* = 108)	*P* value
TC, mmol/L	3.87 ± 0.90	4.25 ± 0.84	4.44 ± 0.95	0.000**
TG, mmol/L	1.47 (1.21, 2.34)	1.26 (0.96, 1.63)	1.40 (1.04, 2.05)	0.037*
HDL-C, mmol/L	0.96 ± 0.18	1.21 ± 0.31	1.18 ± 0.29	0.000**
LDL-C, mmol/L	2.25 ± 0.73	2.44 ± 0.69	2.56 ± 0.78	0.040*
UA, μmol/L	351.1 ± 71.9	312.7 ± 75.2	317.9 ± 70.6	0.004**
Cr, μmol/L	87.6 ± 17.1	74.2 ± 16.0	77.4 ± 13.2	0.000**
FGB, mmol/L	6.1 ± 1.9	5.5 ± 1.3	5.4 ± 1.0	0.007**
HbA1c, %	6.4 ± 1.2	6.0 ± 0.8	6.0 ± 0.8	0.029*
hsCRP, mg/dl	1.10 (0.48, 1.96)	0.67 (0.48, 2.47)	1.15 (0.47, 2.17)	0.839
NTpro-BNP, pg/ml	60.3 (31.4, 119.5)	81.1 (36.7, 156.9)	50.0 (30.0, 87.3)	0.038*

*TC* total cholesterol, *TG* triglyceride, *HDL-C* high density lipoprotein cholesterol, *LDL-C* low density lipoprotein cholesterol, *UA* uric acid, *Cr* creatinine, *FBG* fasting blood glucose, *HbA1C* glycosylated hemoglobin, *hsCRP* high-sensitivity C-reactive protein, *NT-proBNP* N-terminal B-type brain natriuretic peptide precursor. The distribution of TG, hsCRP and BNP was non-normal distribution. The data were expressed as median (quartile 1, quartile 3), i.e. M (p25, p75). *: *P* < 0.05; **: *P* < 0.01

### Comparison of parameters of conventional echocardiography in the three groups

The parameters of conventional echocardiography in the three groups were shown in Table 3. It was showed that there were no significant differences in LAD, LVEDD, LVMI, LVEF, E/A, Sm, E’ and E/E’ in obstructive CAD group, CMD group and control group (*P* > 0.05).

**Table 3:** Comparison of parameters of conventional echocardiography in obstructive CAD group, CMD group and control group

Parameters	obstructive CAD group (*n* = 67)	CMD group (*n* = 67)	control group (*n* = 108)	*P* value
LAD, mm	34.7 ± 3.1	34.3 ± 4.0	34.4 ± 3.8	0.849
LVEDD, mm	47.5 ± 4.0	46.4 ± 4.2	46.4 ± 3.4	0.124
LVMI, g/m^2^	79.5 ± 17.1	78.4 ± 16.7	74.7 ± 16.7	0.112
LVEF, %	70.2 ± 4.2	70.6 ± 4.7	71.1 ± 4.7	0.509
E/A	0.82 ± 0.24	0.88 ± 0.29	0.90 ± 0.29	0.189
E’, cm/s	9.6 ± 2.5	9.8 ± 2.2	10.0 ± 2.3	0.358
E/E’	7.0 ± 2.7	7.2 ± 2.7	7.0 ± 2.4	0.800
Sm, cm/s	10.1 ± 2.2	9.9 ± 1.9	10.0 ± 1.7	0.902

*LAD* left atrial diameter, *LVEDD* left ventricular end-diastolic dimension, *LVMI* left ventricular mass index, *LVEF* left ventricular ejection fraction, *E* early diastolic mitral valve flow velocity, *A* late diastolic mitral valve flow velocity, *E’* early diastolic velocity of left ventricular lateral wall mitral annulus, *Sm* systolic velocity of mitral annulus in left ventricular lateral wall

### Comparison of myocardial strain and SR in the three groups

**Fig. 1:**
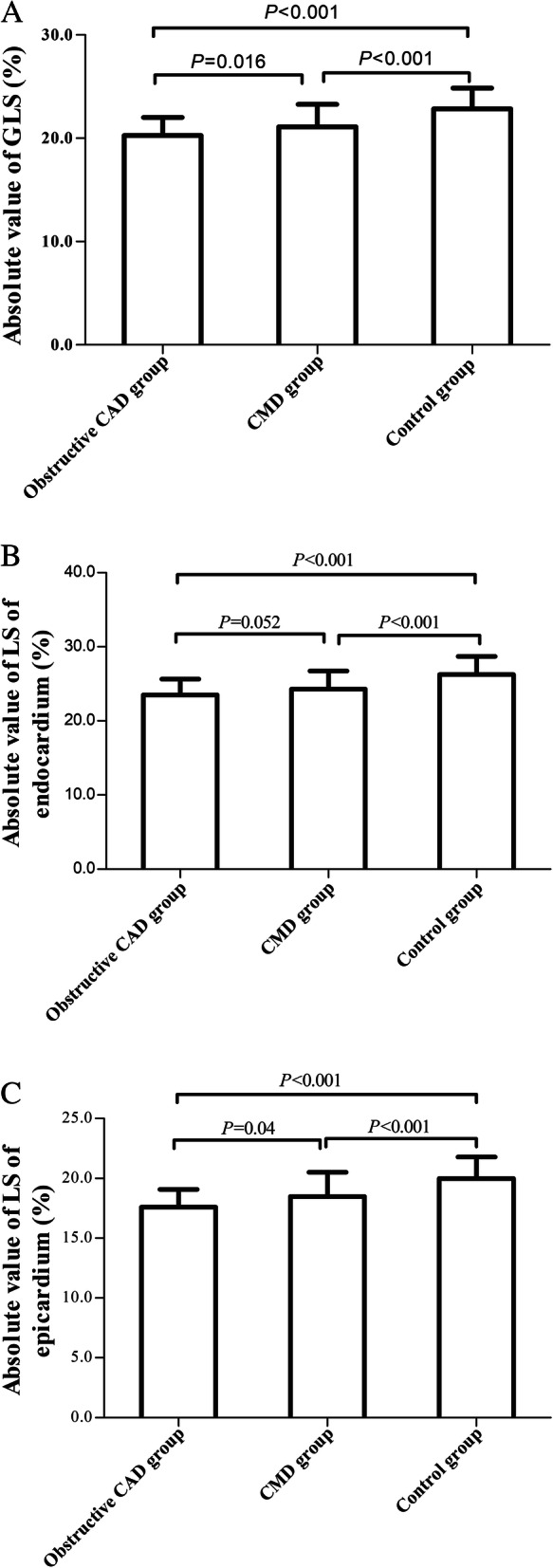
Comparison of GLS, LS in endocardium and LS in epicardium between occlusive CAD group, CMD group and control group. **a** Comparison of GLS between obstructive CAD group, CMD group and control group. **b** Comparison of LS in endocardium between obstructive CAD group, CMD group and control group. **c** comparison of LS in epicardium between occlusive CAD group, CMD group and control group

As shown in Table 4, the GLS in CMD group and obstructive CAD group were significantly lower than that in control group (*P* < 0.05), and the GLS in obstructive CAD group were obviously lower than those in CMD group (*P* < 0.05) (Fig. 1). However, after adjusting for multiple factors such as gender, age, SBP, DBP and complications, the GLS in CMD group and obstructive CAD group were markedly lower than those in control group (*P* < 0.01), while the difference between CMD group and obstructive CAD group disappeared (*P* > 0.05). In addition, the LS of anterior septum, anterior wall, lateral wall, inferior wall and posterior septum in CMD group were markedly lower than those in control group (*P* < 0.05), but there was no significant difference in the LS of posterior wall between CMD group and control group. The LS of each wall in obstructive CAD group were significantly lower than those in control group (*P* < 0.05). The LS of posterior wall in obstructive CAD group were significantly lower than those in CMD group (*P* < 0.05). There was no significant difference in LS of other walls between obstructive CAD group and CMD group (*P* > 0.05).

**Table 4:** Comparison of myocardial strain in obstructive CAD group, CMD group and control group

Parameters	obstructive CAD group (*n* = 67)	CMD group (*n* = 67)	control group (*n* = 108)	*P* value
GLS, %	− 20.3 ± 1.8^△▲^	− 21.1 ± 2.2^△^	− 22.8 ± 2.0	0.000
LS of each ventricular wall, %				
LS of anterior septum	− 17.7 ± 3.3^△^	− 18.5 ± 4.3^△^	−20.8 ± 3.9	0.000
LS of anterior wall	−19.3 ± 3.0^△^	−19.8 ± 3.6^△^	−22.3 ± 2.8	0.000
LS of lateral wall	−21.5 ± 2.5^△^	−22.0 ± 3.3^△^	− 23.9 ± 2.7	0.000
LS of posterior wall	−18.2 ± 2.9^△▲^	− 20.0 ± 3.4	−20.6 ± 2.8	0.000
LS of inferior wall	−20.2 ± 2.9^△^	−20.3 ± 3.2^△^	−22.6 ± 2.8	0.000
LS of posterior septum	−18.5 ± 2.6^△^	−19.3 ± 3.1^△^	−21.0 ± 2.4	0.000
LS of apex	−25.4 ± 3.0^△^	−24.8 ± 3.8^△^	−28.9 ± 3.3	0.000
stratified LS, %				
LS of endocardium	−23.5 ± 2.1^△^	− 24.3 ± 2.5^△^	−26.2 ± 2.4	0.000
LS of epicardium	−17.6 ± 1.5^△▲^	− 18.5 ± 2.0^△^	−20.0 ± 1.8	0.000

*GLS* global longitudinal strain, *LS* longitudinal strain. ^▲^: *P* < 0.05vs. CMD group; ^△^: *P* < 0.05 vs.control group

In terms of stratified strain, the LS of endocardium and epicardium in CMD group and obstructive CAD group were notably lower than those in control group (*P* < 0.05, Fig. 1b and c). However, there was no significant difference in LS of endocardium between CMD group and obstructive CAD group (*P* > 0.05, Fig. 1b). The LS of epicardium in obstructive CAD group were significantly lower than those in CMD group, which were still valid after adjusting for multiple factors (*P* < 0.05, Fig. 1c).

As shown in Table 5, the global longitudinal SR in CMD group and obstructive CAD group was notably lower than those in control group (*P* < 0.05), but there was no significant difference between CMD group and obstructive CAD group (*P* > 0.05). Similarly, the longitudinal SR (anterior septum, anterior wall, lateral wall, posterior wall, inferior wall, posterior septum, apex) in obstructive CAD group were significantly lower than those in control group (*P* < 0.05, Table 5). There was no significant difference in the longitudinal SR of posterior wall between CMD group and control group (P > 0.05), the lonitudinal SR of the other walls in CMD group were lower than those in control group (*P* < 0.05, Table 5). However, the longitudinal SR (anterior septum, anterior wall, lateral wall, inferior wall, posterior septum, apex) in CMD group were significantly lower than those in control group (*P* < 0.05, Table 5). There was no significant difference in longitudinal SR between CMD group and obstructive CAD group (*P* > 0.05, Table 5).

**Table 5:** Comparison of myocardial SR in obstructive CAD group, CMD group and control group

Parameters	obstructive CAD group (*n* = 67)	CMD group (*n* = 67)	control group (*n* = 108)	*P* value
Global longitudinal SR, s^− 1^	−1.25 ± 0.17^△^	− 1.23 ± 0.19^△^	−1.36 ± 0.16	0.000
SR of each ventricular wall, s^−1^				
SR of anterior septum	−1.06 ± 0.22^△^	−1.08 ± 0.26^△^	− 1.19 ± 0.25	0.002
SR of anterior wall	−1.26 ± 0.30^△^	−1.21 ± 0.27^△^	− 1.34 ± 0.21	0.004
SR of lateral wall	−1.31 ± 0.25^△^	−1.28 ± 0.29^△^	− 1.41 ± 0.23	0.002
SR of posterior wall	−1.17 ± 0.24^△^	−1.23 ± 0.24	− 1.25 ± 0.21	0.071
SR of inferior wall	−1.30 ± 0.23^△^	−1.21 ± 0.25^△^	− 1.40 ± 0.20	0.000
SR of posterior septum	−1.13 ± 0.21^△^	−1.12 ± 0.22^△^	− 1.26 ± 0.18	0.000
SR of apex	−1.66 ± 0.39^△^	−1.52 ± 0.48^△^	− 1.86 ± 0.32	0.000

*SR* strain rate. ^△^: *P* < 0.05 vs.control group

### The correlation between CFR and myocardial strain

**Fig. 2:**
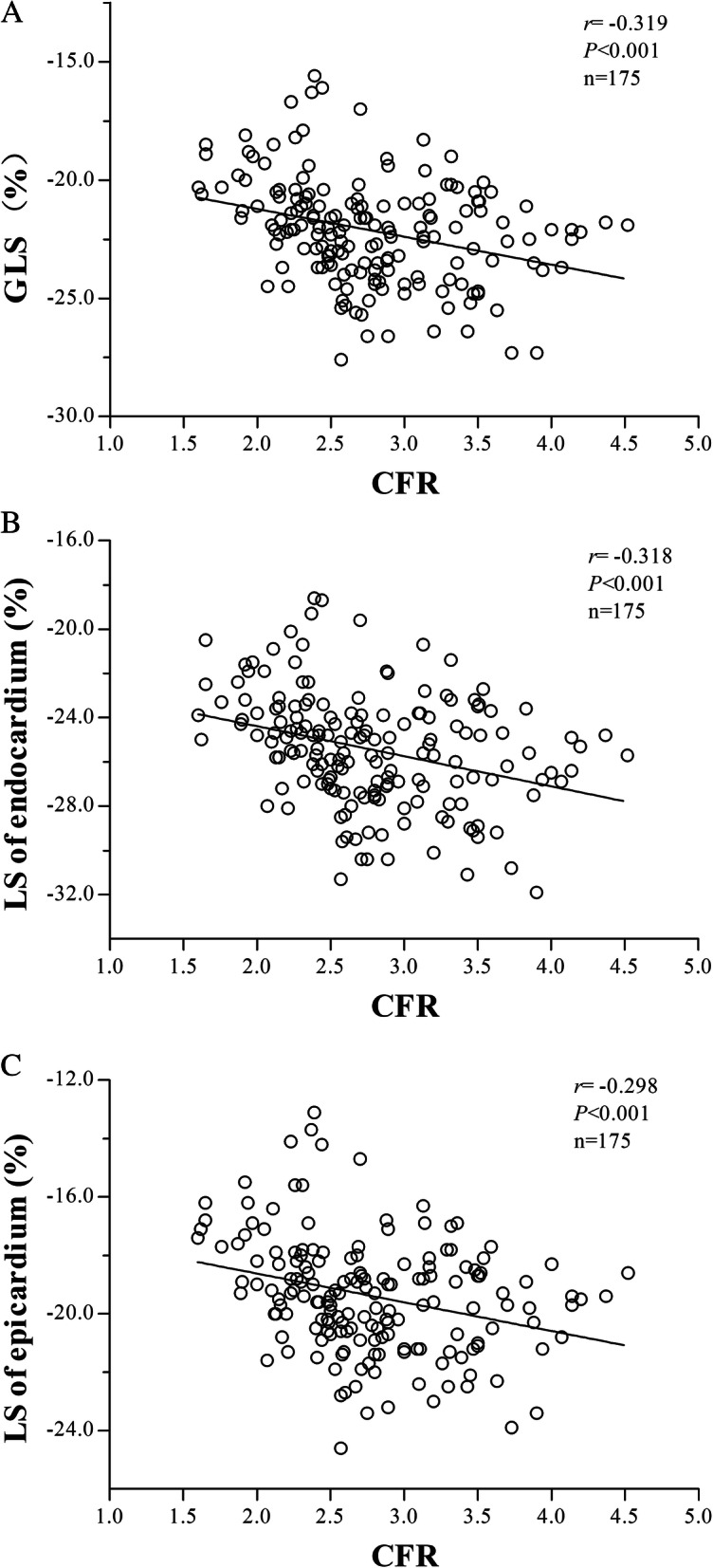
Relevance analysis of GLS, LS in endocardium and LS in epicardium with CFR between CMD group and control group. **a** Scatter plot of correlation analysis between GLS and CFR. **b** Scatter plot of correlation analysis between LS in endocardium and CFR. **c** Scatter plot of correlation analysis between LS in epicardium and CFR

CFR was measured in patients in both CMD group and control group. The correlation between GLS and LS of endocardium and epicardium and CFR was analyzed. As shown in Table 6, GLS, endocardial LS and epicardial LS were negatively correlated with CFR (r = − 0.319, r = − 0.318 and r = − 0.298, respectively) (*P* < 0.01). The scatter plots were shown in Fig. 2a, b and c, respectively.

**Table 6:** The correlation between CFR and myocardial strain

	GLS		LS of endocardium		LS of epicardium	
Parameters	*r*	*P* value	*r*	*P* value	*r*	*P* value
CFR	−0.319	0.000**	−0.318	0.000**	−0.298	0.000**

*CFR* coronary flow reserve, *GLS* global longitudinal strain, *LS* longitudinal strain. **: *P* < 0.01

The values of CFR, sex, age, basic SBP, basic DBP, hypertension, diabetes mellitus, HDL-C, Cr, HbA1c, LVEDD, LVMI, Sm, E/E’ and LVEF were used as independent variables in CMD group and control group and left ventricular GLS was used as dependent variable for covariance analysis. The results showed that CFR was an independent influencing factor of GLS (Table 7).

**Table 7:** Covariance analysis of GLS in CMD group and control group

Parameters	***F***	*P* value
SBP	0.116	0.735
DBP	0.258	0.613
Hypertension	1.155	0.287
Diabetes mellitus	0.056	0.813
HDL-C	0.807	0.373
Cr	0.088	0.768
HbA1c	0.501	0.482
LVEDD	3.581	0.063
E/E’	0.001	0.982
Sm	1.439	0.235
LVMI	0.055	0.816
LVEF	0.977	0.327

*SBP* systolic blood pressure, *DBP* diastolic blood pressure, *HDL-C* high density lipoprotein cholesterol, *Cr* creatinine, *HbA1C* glycosylated hemoglobin, *LVEDD* left ventricular end-diastolic dimension, *LVMI* left ventricular mass index, *LVEF* left ventricular ejection fraction, *E* early diastolic mitral valve flow velocity, *A* late diastolic mitral valve flow velocity, *E’* early diastolic velocity of left ventricular lateral wall mitral annulus, *Sm* systolic velocity of mitral annulus in left ventricular lateral wall

### Predictive effect of myocardial strain on CMD

**Fig. 3:**
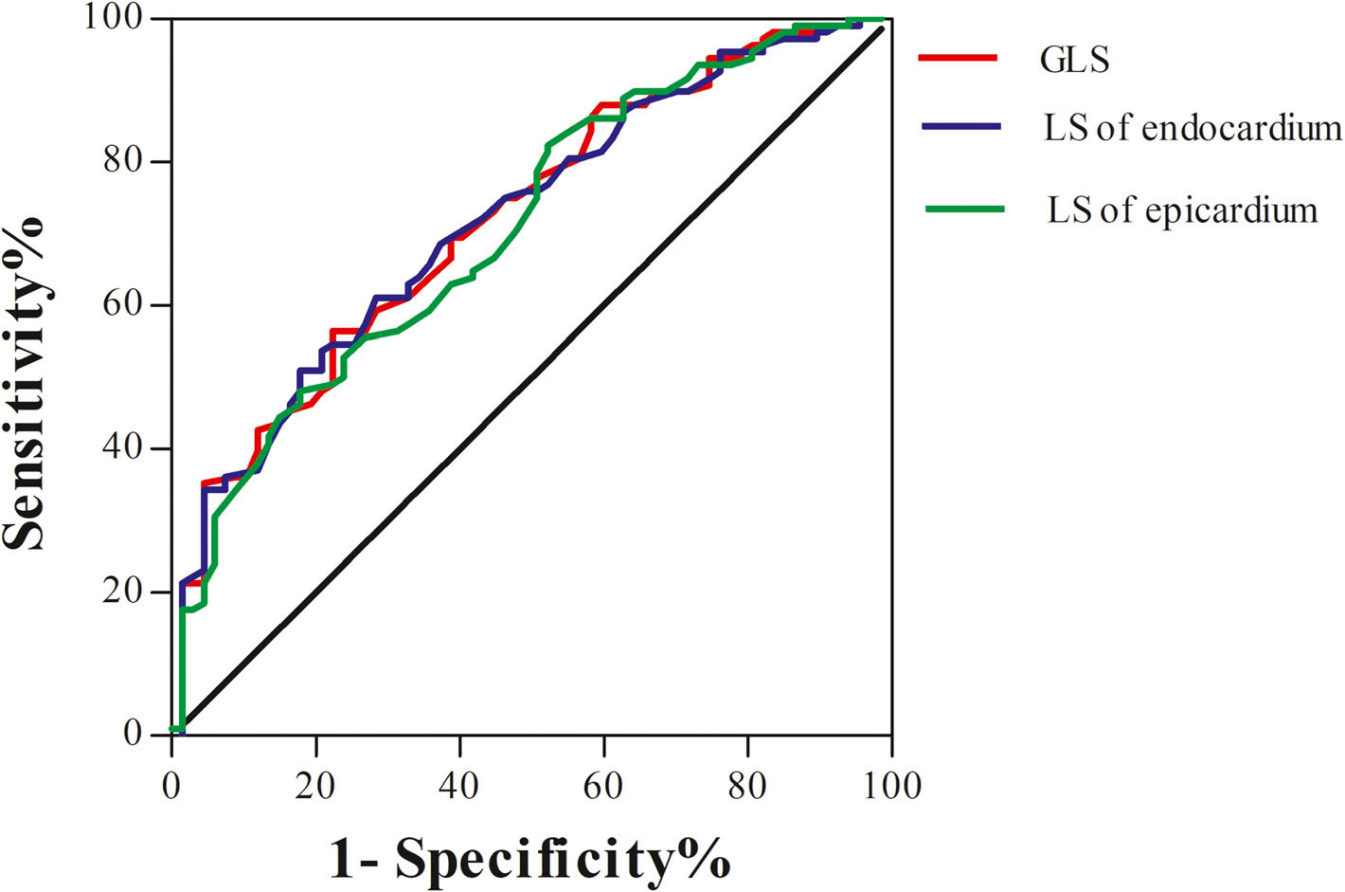
ROC curves of GLS, LS in endocardium and LS in epicardium in diagnosis of CMD

In CMD and control group, the ROC curves of GLS, LS of endocardium and LS of epicardium for CMD diagnosis are shown in Fig. 3. AUC > 0.5 indicates that the index has diagnostic value in diseases. ROC curve showed that GLS = − 22.1% was the cut-off value for diagnosing CMD and the sensitivity and specificity were 68.2 and 61.1%, respectively (AUC = 0.732). Besides, LS of endocardium = − 25.3% was the cut-off value for diagnosing CMD and the sensitivity and specificity were 68.2 and 63.0%, respectively (AUC = 0.731). Furthermore, LS of epicardium = − 19.3% was the cut-off value for diagnosing CMD and the sensitivity and specificity were 62.0 and 63.0%, respectively (AUC = 0.718).

## Discussion

This study compared left ventricular systolic function between patients with CMD and obstructive CAD and normal controls. The results showed that GLS of left ventricle in patients with CMD and obstructive CAD decreased significantly, and there was no statistical difference between the two groups. Furthermore, for non-obstructive CAD patients, GLS was negatively correlated with CFR, and GLS had certain diagnostic value for CMD.

Myocardial ischemia is an important pathological cause of cardiac remodeling, which leads to heart failure. In theory, insufficient perfusion of CMD at the level of myocardial microcirculation can also lead to changes in cardiac structure and function. Sucato [5] and Kato [17] found that coronary microvascular function parameters in patients with HFpEF were significantly lower than patients without heart failure and 76% of patients with HFpEF had a decrease in CFR. An autopsy study also found a significant sparse distribution of coronary microvessels in patients with heart failure [10]. These studies suggest that CMD may be an important pathological mechanism for heart failure. Previous studies have found that CMD does reduce left ventricular diastolic function [18–20]. However, unfortunately, conventional echocardiography is difficult to detect the decline of global left ventricular systolic function represented by LVEF in patients with CMD [21]. Therefore, there are few reports on the effect of CMD on left ventricular systolic function. The maturity of STI technology provides a more sensitive new way to evaluate the systolic function of the heart in patients with CMD, and can detect the impairment of left ventricular systolic function earlier.

In this study, we evaluated left ventricular systolic function by measuring left ventricular LS and SR through 2D-STI technique. The results showed that GLS and SR in CMD group were significantly lower than those in control group. After adjusting for gender, age, complications, biochemical parameters and conventional echocardiographic parameters, there were still significant differences in GLS and SR between the two groups. Some studies have also found that there are varying degrees of decline in the left ventricular strain of patients with or possibly with CMD. Yamur et al. [22] found that the left ventricular GLS was significantly decreased in patients with syndrome X (CSX). Enomoto et al. [23] found that the left ventricular GLS in patients with type 2 diabetes mellitus without obstructive CAD (83% of them were complicated with diabetic microvascular complications) were significantly lower than those in healthy controls, and GLS was significantly correlated with diabetic microvascular complications. There was a high likelihood of CMD among the subjects observed in these studies, such as patients with CSX and diabetic microvascular complications. However, the conclusions of these studies cannot be completely equated with those in patients with CMD due to the lack of quantitative evaluation of coronary microvascular function.

CFR is a common index for evaluating coronary microvascular function. For patients without significant coronary stenosis, reduced CFR suggests the presence of CMD. In our study, TTDE was used to measure the CFR of left anterior descending coronary artery for patients with chest pain and excluding obstructive CAD. CFR < 2.5 was used as the criterion to determine CMD [16], so as to achieve the quantitative evaluation of coronary microvascular function. Our results suggested that the left ventricular systolic function in patients with CMD was decreased while the left ventricular ejection function remained normal. It also suggested that GLS was a more sensitive index for evaluating left ventricular systolic function than LVEF. However, some studies have come to a different conclusion than ours. Michelsen et al. [11] found that the left ventricular GLS reserve in female patients with CMD was significantly lower than that in healthy female, but the study did not obtain statistical differences in GLS between the two groups in resting state, which may be related to the differences in the population and research methods included in our study.

The results of myocardial stratification strain showed that the endocardial LS and epicardial LS in CMD group were significantly lower than those in control group. Compared with the epicardium, the subendocardial microvasculature bears higher wall tension, so the subendocardial perfusion first decreases during myocardial ischemia. The subendocardial myocardium is dominated by longitudinal myocardial fibers, so the decrease of LS is often observed earlier in the endocardial myocardium [24]. When the myocardial involvement was aggravated, the whole wall LS decreased. In this study, the decrease of the endocardial LS and epicardial LS in CMD group were significantly lower than that in control group, suggesting that myocardial ischemia in patients with CMD can lead to the decrease of left ventricular wall strain.

Meanwhile, GLS, endocardial LS and epicardial LS were negatively correlated with CFR. After adjusting for several factors, multivariate analysis showed that CFR was an independent factor of GLS. This was consistent with the results of Ikonomidis et al. [25], which found that CFR was negatively correlated with left ventricular GLS in patients with untreated hypertension without obstructive CAD. It means that the lower the CFR, the worse the longitudinal deformability of myocardium, suggesting that the decrease of left ventricular strain may be related to the decrease of CFR.

Epicardial coronary artery stenosis or occlusion can cause left ventricular regional or global systolic dysfunction. However, in the early stage of disease, these abnormalities are often difficult to observe by conventional echocardiography. At this time, STI technology shows a good diagnostic value in the early stage. A series of previous studies have shown that the left ventricular strain parameters in patients with obstructive CAD have decreased in varying degrees, which is related to the degree of coronary artery stenosis, the number of lesion branches and the presence of collateral circulation [26–28]. Left ventricular strain parameters can accurately locate the lesion site of myocardial infarction, determine the infarct size and the degree of infarct wall penetration [29, 30]. Left ventricular strain can be used as an assistant tool in diagnosing obstructive CAD, and it can also predict the degree of coronary artery stenosis [31–35]. Our results showed that GLS and its SR, left ventricular wall LS and its SR, endocardial LS and epicardial LS in patients with obstructive CAD were significantly lower than those in control group. It suggests that the left ventricular systolic function has been impaired in patients with obstructive CAD, even though no abnormal wall motion has been found by conventional echocardiography and LVEF remains in normal range. This further confirms the value of 2D-STI in evaluating left ventricular systolic function in patients with obstructive CAD.

CMD and obstructive CAD cause coronary microcirculation and epicardial coronary ischemia respectively. Whether there are differences in the degree of impairment of cardiac systolic function between them is unknown. Comparing the strain parameters in CMD group with those in obstructive CAD group, we found that the GLS, endocardial LS and epicardial LS in obstructive CAD group were lower than those of CMD group. However, after adjusting for multiple factors, the GLS and stratified LS of left ventricle in CMD group and obstructive CAD group had no statistical difference, which indicated that the degree of left ventricular systolic function impairment in the two groups was similar.

Myocardial ischemia in patients with angina pectoris or objective evidence of myocardial ischemia, cannot be completely excluded even if coronary angiography shows no or mild stenosis (stenosis < 50%). CMD is one of the main causes of myocardial ischemia in patients with non-obstructive CAD. Research shows that about 51% of men and 54% of women with suspected CAD have CMD [36]. This study found that left ventricular strain decreased in patients with CMD. Can this decrease be used to diagnose CMD? ROC curve analysis showed that GLS and stratified LS had better diagnostic value for CMD, among which GLS had the highest diagnostic value. Endocardial LS and epicardial LS also have certain diagnostic value for CMD. Our results suggest that GLS and stratified LS may be an assistant method for screening CMD in patients with chest pain without obstructive CAD.

The main limitation of this study is that it is a retrospective study, and the sample sizes of obstructive CAD group and CMD group are small, which may lead to some bias in the research results. Patients with obstructive CAD cannot completely exclude the presence of coronary microcirculation disorders, which may have a certain impact on the results of the study.

## Conclusion

Left ventricular LS and SR were significantly decreased in patients with CMD and obstructive CAD, and there was no statistical difference between the two groups, suggesting that early left ventricular systolic dysfunction existed in both groups with similar degrees. In addition, GLS was negatively correlated with CFR in patients with CMD, and CFR was an independent influencing factor of GLS. GLS and stratified LS have certain diagnostic value for CMD.

## Data Availability

Not applicable.
